# Annual Commencements of the University of Maryland Dental Department

**Published:** 1885-02

**Authors:** 


### Editorial, Etc.

---

Annual Commencement of the University of Maryland Dental Department.—The Third Annual Commencement of this Dental School, in connection with the Seventy-eight Annual Session of the University of Maryland, was held March 17th, 1885, at the Academy of Music, Baltimore. The exercises were opened with prayer by the Rev. Culbreth B. Perry, and after the reading of the Mandamus, the names of the dental graduates, thirty-six in number, were announced by Prof. Ferdinand J. S. Gorgas, Dean of the Dental Department.

The degree of "Doctor of Dental Surgery" was then conferred upon the following gentlemen, by Prof. Geo. W. Miltenberger who acted for the Provost of the University, the Hon. S. Teackle Wallis, L. L. D., who was absent on account of illness :

Bailey, Madison A. ........ South Carolina
Beadles, E. Payson ........ Virginia
Bradford, Henry Clinton ........ Virginia
Brown, Claude D ........ Virginia
Carlisle, John P ........ South Carolina
Carter, Joseph W ........ Missouri
Comegys, Thomas M ........ Tennessee
Cooke, Frank. J ........ Texas
Dorset, Willie Edward ........ Virginia
Fournier, Jr., Joseph ........ New York
Groshans, Ferdinand ........ Maryland
Hebbel, Charles W ........ Maryland

Helm, John W. .......................................... Maryland
Hill, Charles E. .......................................... Australia
Hougland, Ulysses Sylvester, .......................................... Indiana
Howland, Clarence Henry .......................................... D. Columbia
Howlett, A. Hersey .......................................... Pennsylvania
Hundley, Peyton .......................................... Virginia

Josselyn, M. D. Eli E. .......................................... New Brunswick
Kloeber, John S. .......................................... Virginia
Matthews, Augustus .......................................... North Carolina
McQuown, Robert T. .......................................... Virginia
McQuown, William P. .......................................... Virginia
Norwood, Wm. McIntosh .......................................... S. Carolina
Parker, Will W. .......................................... Minnesota
Pitts, Henry Clay .......................................... North Carolina
Perkins, Capers D. .......................................... Georgia

Ranson, Jr., James M. .......................................... West Virginia
Rutledge, Brooks .......................................... S. Carolina
Trapp, Wm. Sherman .......................................... Pennsylvania
Twitchell, Fred. A. .......................................... Minnesota
Wangemann, Albert .......................................... Germany
Welch, Floyd J. .......................................... Virginia
Wegge, William F. .......................................... Wisconsin
Wood, Frank Le Roy .......................................... Maine

The "University Pledge" relating to empiricism, was assented to by every graduate. The following prizes were then awarded to members of the Graduating Class: The beautifu "*University Gold Medal* to William F. Wegge, of Wisconsin, for the highest nnmber of votes at the examinations for the degree of "Doctor of Dental Surgery." *Honorable mention*, for second highest number John S. Kloeber, of Virginia.

*The S. S. Prize*—"Dental Engine," to Joseph Fournier, Jr., of New York, for the best two fillings, one of cohesive and one of non-cohesive gold. *Hon. mention*: Peyton Hundley, of Virginia.

*The Snowden & Cowman Prize.* Set of Chapin A. Harris Forceps" to William McIntosh Norwood, of South Carolina, for the best full upper set of gum teeth on metal. *Hon. mention*: Joseph Fournier, Jr., of New York.

*The S. S. White Prize*: Set of Varney's Pluggers," to Joseph Fournier, Jr., of New Yo.k, for the best partial upper set of gum teeth on metal.

*The James H. Harris Prize*: "Gold Medal" to Brooks Rutledge, of South Carolina, for the best nou-cohesive gold filling. *Hon mention*: John P, Carlisle, of South Carolina.

*The B. M. Wilkerson Prize*: "Gold Medal," to Joseph Fournier, Jr,, of New York, for the best continous-gum set.

*The R. Y. Henley Prize*: "Gold Medal to Joseph Fournier, Jr., of New York, for the best combinatiou set of celluloid and metal with Soldered rim. •

*The Chas. L. Steel Prize*: "Gold Medal," to Frank Le Roy Wood, of Maine, for the best cohesive gold filling. *Hon. mention*: Thos. M. Comegys, of Tennessee.

*The Jno. C. Uhler Prize*: "Gold Medal," to Clarence H. Howland, of District of Columbia, for best Celluloid set.

*The Blake & Co. Prize*: "Taylor Automatic Mallet" to Brooks Ruutledge, of South Carolina, for best gold filling in specimen tooth.

*The B. H. Catching Prize*: "Southern Dental Journal," to Floyd J. Welch, of Virginia, for best examination on Dental Mateiia Medica and Therapeutics."

The Valedictory Address was delivered by Prof. R. Dorsey Coale, Ph. D. and was replete with excellent advice to tl'e graduates and was attentively listened to by the large audience present. The exercises closed with the benediction.

The number of matriculates for the Session of 1884-'85, was seventy-four, all of whom were dental students from every part of this country, Germany, Belgium, France, Australia, Mexico, New Brunswick, and Brazil.

The Annual meeting of the Alumni of this Dental Deparfment was held on the evening of commencement day, and was presided over by Dr. R. D. Dodson, the Vice-President—owing to the absence of the President Dr. Hungerford, on account of illness. The Alumni Banquet took place at the Howard House at the close of the meeting, and was enjoyed by a large number of prominent dentists in connection with the graduating class.

The "claas contest" for Prizes was held March 14th, 1885. in the Infirmary and was a very interesting occasion, occupying the entire day. The following gentlemen acted as committees in making the awards: Drs. George B. Steel, of Va.. William S. McDowell, of Md., E. B. Bliss, of D C.. Geo. H. Chewning, of Va., R. Y. Henley, of Va., A. C. McCurdy, of Md., R. D. Dodson, of Pa., L. C. Tucker, of Va., Wm. R. Renalds, of Va,, M. G. Sykes, of Md., B. M. Hopkinson, of Md., and D. Genese, of Md., all of whom were greatly pleased with the proficiency exhibited by the competing students.

